# Study of the Anticancer Potential of Plant Extracts Using Liver Tumor Microphysiological System

**DOI:** 10.3390/life12020135

**Published:** 2022-01-18

**Authors:** Hafiz Muhammad Umer Farooqi, Anupamma Sammantasinghar, Farzana Kausar, Muhammad Awais Farooqi, Abdul Rahim Chethikkattuveli Salih, Kinam Hyun, Jong-Hwan Lim, Atif Ali Khan Khalil, Abdul Samad Mumtaz, Kyung Hyun Choi

**Affiliations:** 1 Department of Mechatronics Engineering, Jeju National University, Jeju-si 63243, Korea; umerfarooqi@jejunu.ac.kr (H.M.U.F.); anupamasamantasinghar31@gmail.com (A.S.); awaisfarooqi@stu.jejunu.ac.kr (M.A.F.); abdul.rahim350@gmail.com (A.R.C.S.); gusrlaska8204@jejunu.ac.kr (K.H.); jhlim@jejunu.ac.kr (J.-H.L.); 2 National Control Laboratory for Biologicals, Drug Regulatory Authority of Pakistan, Islamabad 44090, Pakistan; 3 Department of Plant Sciences, Quaid-i-Azam University, Islamabad 45320, Pakistan; kausaufarzana4915@gmail.com; 4 Department of Biological Sciences, National University of Medical Sciences, Rawalpindi 46000, Pakistan; atif.ali@numspak.edu.pk; 5 BioSpero, Inc., Jeju-si 63243, Korea

**Keywords:** liver tumor microphysiological system, *Acer cappadocicum Gled*, plant extracts, anticancer activity, transepithelial electrical resistance sensor

## Abstract

Background: Plants have been considered a vital source of modern pharmaceutics since the paleolithic age. Contemporary chemotherapeutic drugs for cancer therapy are chemical entities sourced from plants. However, synthetic drugs or their derivatives come with severe to moderate side effects for human health. Hence, the quest to explore and discover plant-based novel anticancer drugs is ongoing. Anticancer activities are the primary method to estimate the potential and efficacy of an extract or compound for drug discovery. However, traditional in vitro anticancer activity assays often show poor efficacy due to the lack of in-vivo-like cellular environment. In comparison, the animal-based in vivo assays lack human genetic makeup and have ethical concerns. Aim: This study aimed to overcome the limitations of traditional cell-culture-based anticancer assays and find the most suitable assay for anticancer activity of plant extracts. We first reported utilizing a liver tumor microphysiological system in the anticancer effect assessment of plant extracts. Methodology: Methanolic extracts of *Acer cappadocicum Gled* were used to assess anticancer activity against liver tumor microphysiological system (MPS), and cell viability, liver function tests, and antioxidant enzyme activities were performed. Additionally, an embedded transepithelial electrical resistance sensor was utilized for the real-time monitoring of the liver tumor MPS. The results were also compared with the traditional cell culture model. Results: The study demonstrated the superiority of the TEER sensor-based liver tumor MPS by its better anticancer activity based on cell viability and biomarker analysis compared to the traditional in vitro cell culture model. The anticancer effects of the plant extracts were successfully observed in real time, and methanolic extracts of *Acer cappadocicum Gled* increased the alanine transaminase and aspartate aminotransferase secretion, which may reveal the different mechanisms of these extracts and suggest a clue for the future molecular study of the anticancer pathways. Conclusion: Our results show that the liver tumor microphysiological system could be a better platform for plant-based anticancer activity assessment than traditional cell culture models.

## 1. Introduction

Chronic liver diseases result in two million deaths worldwide each year. At the same time, liver cancers are the fourth most invasive cancer globally [1]. Viral hepatitis, alcohol abuse, metabolic syndromes, sedentary lifestyle, liver cirrhosis, and hepatocellular carcinoma are constant pressure-triggering elements for hepatic disease prevalence [2]. Early screening of hepatic diseases, vaccination, and antiviral treatments have substantially reduced liver ailments, but the lack of efficient in vitro models could be one of the main hurdles in discovering new therapies for hepatic carcinomas [3,4]. The animal cancer model is commonly used for efficacy and safety testing of anticancer drugs. However, this method is expensive, laborious, and proven to be misleading due to cross-species genetic variations and poses ethical concerns [5]. Traditional in vitro cell culture cancer models are convenient and can be operated in high-throughput processes. Still, they do not mirror the complex pathophysiological functions of human tissue and organs due to lack of shear stress, cellular crosstalk, extracellular matrix (ECM) composition, histology, etc. [6]. Humanized microphysiological systems (MPS) are based on cutting-edge microfluidic technology, translating tissue-specific vital features of human organs and their in vivo interplay. Hence, MPS can pave a path for a better understanding of a medicinal agent’s efficacy and toxicity studies than any conventional cell culture model. MPS serves as the organ-on-chip system for recapitulating the tissue or tumor microenvironment to analyze a compound or drug’s effect just like an in vivo environment [7]. Moreover, MPS are acting as a preclinical testing tool to reduce the use of animal models. The pathophysiological aspects of the microfluidic environment can also be validated by the monitoring of cell-to-cell tight junction proteins and extracellular composition [8]. Transepithelial electrical resistance (TEER) and electrical cell–substrate (ECIS) measurements are emerging as an alternative to laborious conventional molecular and biochemical assays as they are quick and noninvasive [9]. TEER applicability ranges from cell monolayer-barrier integrity measurement, cell-to-cell tight junction estimation, extracellular matrix (ECM) quantification, and drug toxicity testing [8,10,11]. However, TEER sensing has not been employed to monitor the effect of plant extracts in a tumour microphysiological system to elucidate its application in the field of plant-extract-based anticancer drug screening.

Thousands of unidentified plant species are an untapped source of natural metabolites responsible for high anticancer potential, and can be characterized by biological activities and spectroscopic methods. Much attention has been given to the plant and herbal extracts possessing various biochemical constituents to inhibit or kill the cancerous cells due to their natural source [12,13,14]. Around 80% of the underdeveloped countries rely on plants for therapeutic purposes [15]. Indigenous Himalayan inhabitants reported ethnobotanical uses of *Acer cappadocicum Gled* against several diseases, such as rheumatoid arthritis, fatty liver disease, conjunctivitis, and inflammation. Additionally, several bioactivity analyses have confirmed the ethnomedical significance of Acer species towards hepatoprotective function [16,17,18,19,20,21]. Previously, the study of the phytochemical evaluation, antimicrobial activities, and cytotoxicity of *Acer cappadocicum Gled* extracts forecasted their potential for drug discovery [19]. Hence, the methanolic extracts of *Acer cappadocicum Gled* were chosen to evaluate further the anticancer potential of *Acer cappadocicum Gled* using a liver tumor MPS.

The primary objective of the present study was to utilize a liver tumor MPS for testing the anticancer activities of plant extracts and their comparison with the traditional cell culture model. The anticancer activity of methanolic extracts of *Acer cappadocicum Gled* was tested against the liver tumor MPS composed of hepatocellular carcinoma cells. Various concentrations of the methanolic extracts of *Acer cappadocicum Gled* were perfused through the cell culture media. A chip-embedded TEER sensor was applied to evaluate the impact of the plant extracts on the cell–cell tight junctions. The study was also translated with a traditional in vitro cell model (96-well cell culture plate), and a considerable difference among the anticancer activities was noted by measuring various biomarkers.

## 2. Materials and Methods

### 2.1. Fabrication of Microfluidic Device

The glass-based microfluidic chip has consisted of two glass slides (soda–lime–silica glass, 1.1 mm thick, 56 mm long, and 41 mm wide), which were stacked on each other. A biocompatible microfluidic channel separated the two glass slides to create a single chip. The biocompatible microfluidic channel was 3D printed using a multi-head 3D printer, and polydimethylsiloxane (PDMS) (Dow SYLGARD^®^, Dow Corning, Rochester, NY, USA) was used as a substrate. Before loading glass chips into the 3D printer stage (channel height 300 µm, width 800 µm), a custom-built magnetic chip holder supported the microfluidic glass chip assembly. Silicon gaskets were employed in the magnetic chip holder to avoid fluid leakage.

### 2.2. Cell Culture and Seeding on the Microfluidic Chip

**Figure 1:**
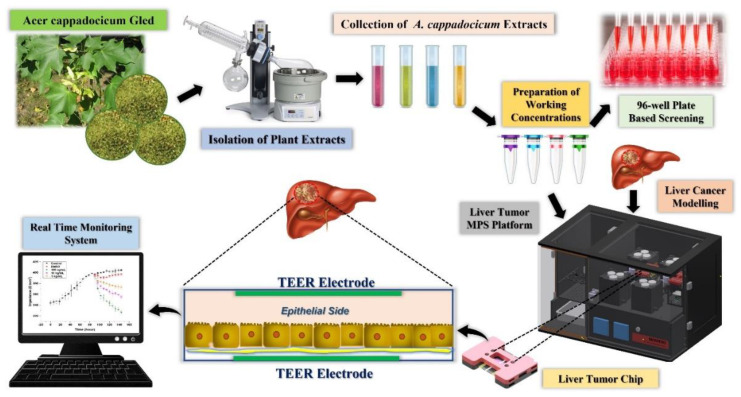
The plant extract preparation method and liver tumor MPS platform.

An immortal human-origin HepG2 hepatocellular carcinoma cell line was purchased from Korea Cell Line Bank (Seoul, Korea). HepG2 cells were cultured in high-glucose Dulbecco’s modified eagle cell culture medium (DMEM) (catalog# 11995040. ThermoFisher Scientific, Waltham, MA, USA) supplemented with 5% fetal bovine serum (FBS) *v/v* (catalog # 16000044, ThermoFisher Scientific, Waltham, MA, USA) and 1% *v/v* penicillin and streptomycin (P&S) antibiotic solution for cell culture (catalog # 15070063, ThermoFisher Scientific, Waltham, MA, USA). The cellular culture was maintained in a cell culture incubator (humidified at 37 °C, 5% CO_2_). The expansion of HepG2 cells was carried out by passing three times before seeding on the microfluidic chip. Dulbecco’s phosphate-buffered saline (DPBS) (catalog # 14190144, ThermoFisher Scientific, Waltham, MA, USA) was warmed in a water bath at 37 °C for washing the cells to wash out the metabolic wastes and debris. The cell culture was trypsinized when it achieved 90% confluency with 0.50% trypsin EDTA solution (catalog # 25300054, ThermoFisher Scientific, Waltham, MA, USA). The microfluidic glass chips were cleaned with 90% isopropyl alcohol and rinsed thrice with double-distilled water in a biosafety cabinet. After that, the chips were air-dried and UV irradiated for 60 min to achieve sterilization. A customized magnetic extracellular matrix (ECM) and cell seeding kit (M-Physio™ Seeding Kit, BioSpero Inc., Jeju-si, South Korea) was utilized to apply ECM and for cell seeding on the cell culture area of the microfluidic glass chip. Fibronectin (ThermoFisher Scientific, Waltham, MA, USA) solution was prepared in double-distilled water at the concentration of 25 µg/mL for the hepatocellular carcinoma cell attachment to the surface of the microfluidic glass chip. Cells were seeded at the concentration of 400,000 cells/mL in DMEM through the magnetic cell seeding kit and were allowed to attach to the ECM surface for 6 h in a cell culture incubator (humidified at 37 °C, 5% CO_2_). After that, the cell culture media was removed, and the top and bottom glass slides of the microfluidic chip were assembled in the custom-built magnetic chip holder (M-Physio™ Chip Holder, BioSpero Inc., Jeju-si, South Korea). The assembled microfluidic chip was positioned in a microfluidic platform (M-Physio™ MPS Platform, BioSpero Inc., Jeju-si, South Korea) to form a cancer tissue monolayer in a dynamic cell culture microenvironment, as represented in Figure 1. The microfluidic chip was connected to the cell culture media reservoir (5 mL) through the microfluidic tubing. A microfluidic peristaltic pump was set up at the speed of 60 µL per minute to create the shear stress of 0.5 dyn/cm^2^ in the microfluidic cell culture channel. The shear stress was calculated by the equation given below:

*τ* = 6μQ/wh^2^

where “µ” represents the viscosity of the cell culture media, “Q” signifies the flow rate of the cell culture media, and “w” exhibits the width of the channel. At the same time, “h” stands for the height of the microfluidic channel.

### 2.3. Collection and Preparation of Plant Extracts

*Acer cappadocicum Gled* sample (branches and green leaves) was collected from the lesser Himalayas (Pallas Vale, Kohistan, Pakistan) and prepared as described previously [19]. Briefly, after the plant authentication, extracts were performed through mechanical maceration. The dry leaves and the plant branches were ground in a mill (mesh number 60), and then soaked in the solvent (20 g/200 mL). The solution was shaken for 72 h in an automated shaker. Whatman Number 1 filter was used to filter the solution. The solution was placed under a shade at room temperature for the evaporation of the solvent and extraction of the crude extracts. The crude extract was then suspended in H_2_O and partitioned with methanol. The working concentration (100 ng/mL, 10 ng/mL, 1 ng/mL) of the crude extracts was prepared in dimethyl sulfoxide (DMSO) and stored at 4 °C in the dark.

### 2.4. Live/Dead Assay and ROS Estimation Assay

The cells were washed thrice with Dulbecco’s phosphate-buffered saline (DPBS) solution. A live/dead assay kit (catalog # 15070063, ThermoFisher Scientific, Waltham, MA, USA) was used to stain the cells by following the manufacturer’s instructions. Cellular Reactive Oxygen (ROS) Assay Kit (catalog # ab113851, Abcam, Waltham, MA, USA) was used to stain the cells for 45 min. After the staining procedures, the cell culture area of the microfluidic chip was rinsed with DPBS, and a mounting media (Fluoromount Aqueous Mounting Media, Sigma-Aldrich, St. Louis, MO, USA) was used to place the coverslip on the tissue. At the same time, CellTiter 96^®^ AQ_ueous_ One Solution Cell Proliferation Assay (MTS) Kit (catalog # G3581, Promega, Madison, WI, USA) was utilized for MTS assay. The confocal imaging reader (Cytation C10, BioTek, Winooski, VT, USA) was used at the excitation wavelength (530–560) and emission wavelength (530–645) to obtain confocal micrographs. The fluorescent images were processed using ImageJ software (Version 1.52 p, NIH, Bethesda, ML, USA).

### 2.5. Biomarker Analysis

ALT, AST, urea, albumin, and SOD assays were performed to estimate the impact of plant extracts on the liver tumor MPS. Alanine Transaminase Activity Assay Kit (catalog # ab105134, Abcam, Waltham, MA, USA), Aspartate Aminotransferase Activity Assay Kit (catalog # ab105135, Abcam, Waltham, MA, USA), Urea Assay Kit (catalog # ab83362, Abcam, Waltham, MA, USA), Human Albumin ELISA Kit (catalog # ab179887, Abcam, Waltham, MA, USA), and Superoxide Dismutase Activity Assay Kit (catalog # ab65354, Abcam, Waltham, MA, USA) kits were used for ALT, AST, urea, albumin, and SOD quantification, respectively. Cell culture media samples were briefly taken at specific time intervals and immediately stored at −80 °C. In contrast, an SOD assay was performed on the cell lysate prepared by the procedure previously described. Before the biomarker estimation procedure, cell culture media samples were thawed in a water bath at 37 °C. A semi-automated microplate reader (SpectraMax iD3, Molecular Devices, Silicon Valley, CA, USA) was utilized to take the readings per the manufacturer’s instructions.

### 2.6. Statistical Analysis

For the validation of the results, TEER, 96-well, and MPS-based viability studies were performed from different positions of the chip, and the relative light unit (RLU) was calculated multiple times from live/dead assay confocal micrographs. In addition, a one-way analysis of variance (ANOVA) test was performed using Tukey’s honestly significant difference (HSD) procedure to verify the statistical significance of the data, which facilitated pairwise comparisons within the acquired data. For statistical comparisons, a *p*-value ≤ 0.05 was considered significant versus the lowest value and denoted by “*”.

## 3. Results

### 3.1. Real-Time Monitoring of Liver Tumor MPS

The liver tumor MPS was established, as shown in Figure 1, and the real-time monitoring of the MPS was performed for six days using a chip-embedded TEER sensor. In vivo physiochemical conditions aid cells in propagating and maintaining their molecular cues. The characterization of cell morphology and differentiation occur due to the fluidic shear stress present in the living bodies [22]. Conventional cell culture models do not offer peculiar share stress or the controlled microenvironment required by cells for their healthy propagation. Physio-mechanical components of MPS steer the microenvironment by applying shear stress [23]. It has been previously shown that the shear stress of 0.5 dyn/cm^2^ yields a superior monolayer in an MPS compared to the stationary cell culture systems [10]. The tumor MPS monitored TEER values with six-hour intervals. An increase in the TEER values was observed in the control experiments, which depicts the cell’s propagation, differentiation, cell-to-cell tight junction formation, and, eventually, a tumor monolayer formation. The present study reveals the TEER range of 343–392 Ω/mm^2^ for the formation of a compact tumor monolayer. The results follow a previous study where the TEER range of 345–395 Ω/mm^2^ was the value for monolayer formation [10]. It has been proven that a continuous supply of FBS and cellular differentiation results in a consistent increase in TEER, and the same phenomenon was observed in the current study [8]. DMSO is widely used in biomedical sciences and is considered one of the best universal solvents. The ready dissolution of plant-based extracts made it an ideal interplay medium between the cells and the chemical molecules [24,25]. Jang et al. claimed that DMSO affects the cell-to-cell tight junction protein expression and physiology [26]. However, it was found that DMSO negatively affected the TEER values with a liver tumor MPS, and the TEER value dropped to 397 Ω/mm^2^ on the six days, while the control tumor MPS presented the TEER value of 404 Ω/mm^2^ for the same period. The biomarker release and cell viability data of tumor MPS further strengthen the argument that even the minor quantities of DMSO significantly impact the liver tumor MPS. Three concentrations (100 ng/mL, 10 ng/mL, and 1 ng/mL) of the methanolic extracts of the *A. cappadocicum* were applied to the tumor MPS on the third day of the experiment, and their effect on the liver tumor MPS was observed for three days. The highest concentration of the extracts significantly decreased the TEER value up to 327 Ω/mm^2^, which signifies the cell-to-cell tight junction disruption. Similarly, other concentrations of the plant extracts also resulted in a drop in TEER. Farzana et el. reported the presence of quinones in the *A. cappadocicum,* which are known to disrupt the barrier integrity in cancer epithelial cells, interfering with the cellular transcriptional regulation using the histone deacetylase (HDAC) [27]. Furthermore, quinones are also known to boost reactive oxygen species (ROS), which are a proven source of tight junction protein degradation. ROS release was directly proportional to cell tight junction damage and lower than usual TEER values in a previous study [19,28].

### 3.2. Effect of A. cappadocicum on Liver Function Tests

**Figure 2:**
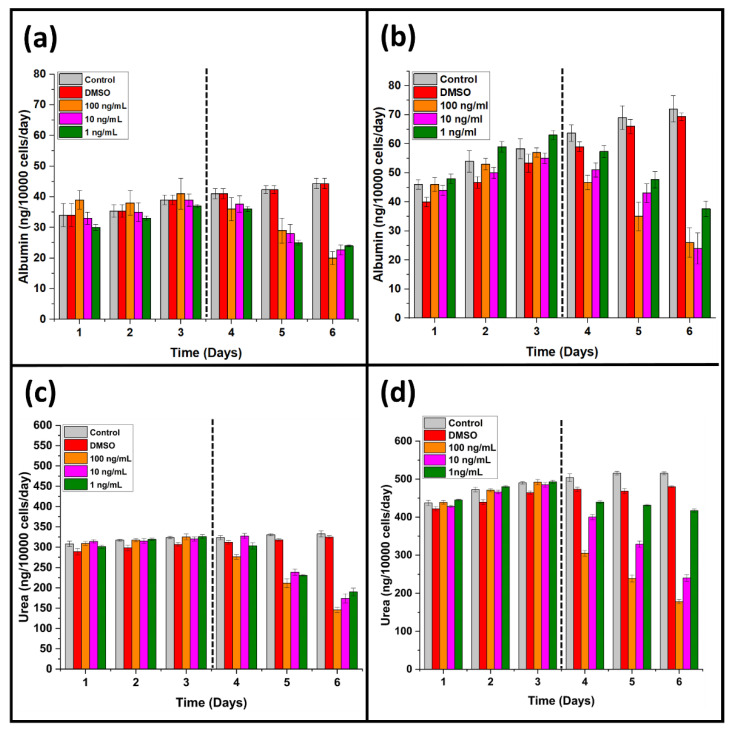
Comparison of the liver tumor MPS with the traditional 96-well plate cell culture model. The cell culture models were treated with plant extracts after 3 days of stable culture. (**a**) Illustration of albumin production in the 96-well plate cell culture model before and after the treatment with plant extracts. (Dotted line is divining the pretreatment and post-treatment biomarker yield.) (**b**) Illustration of albumin production in the liver tumor MPS model before and after the treatment with plant extracts. (Dotted line is divining the pretreatment and post-treatment biomarker yield.) (**c**) Illustration of urea release in the 96-well plate cell culture model before and after the treatment with plant extracts. (Dotted line is divining the pretreatment and post-treatment biomarker yield.) (**d)** Illustration of urea release in the liver tumor MPS model before and after the treatment with plant extracts. (Dotted line is divining the pretreatment and post-treatment biomarker yield.)

Liver function tests, such as albumin, urea, ALT, and AST, are the set of biomarkers representing the overall pathophysiological state of the liver-specific cells. Hepatocellular carcinoma cells continuously produce liver-specific proteins and other biomarkers, which can be utilized to monitor the effect of an anticancer agent [29,30]. MPS is known to influence the yield of albumin by HepG2 cells greatly. Comparing the conventional cell culture model (96-well cell culture plate), liver tumor MPS showed a considerable difference in albumin release between the two cell culture models [10,31]. The albumin production by liver tumor MPS was onefold increased compared to the conventional cell culture model (Figure 2a,b). A similar response was observed in the case of urea release by the liver tumor MPS shown in Figure 2b,c.

On the other hand, the application of *A. cappadocicum* extracts significantly reduced the albumin and urea release from the hepatocellular carcinoma cells. A threefold decrease was noted in albumin production with the 100 ng/mL plant extract, while the same extract concentration reduced the urea release by twofold (Figure 2). In addition, the conventional cell culture model observed a related urea and albumin release. Interestingly, there were considerable differences found between the biomarker yield by the liver tumor MPS and the traditional cell culture model.

**Figure 3:**
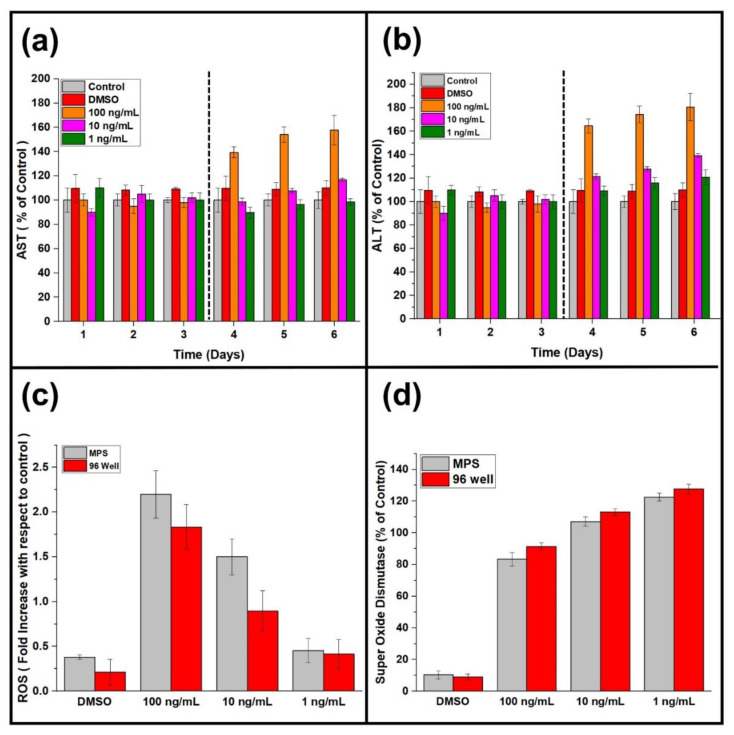
Evaluating the anticancer effect of plant extracts through cancer cell damage indicators (ALT, AST, ROS, and SOD). (**a**) AST release after treatment with DMSO and plant extracts (100 ng mL^−1^, 10 ng mL^−1^, 1 ng mL^−1^). (Dotted line is divining the pretreatment and post-treatment biomarker yield.) (**b**) AST release after treatment with DMSO and plant extracts (100 ng mL^−1^, 10 ng mL^−1^, 1 ng mL^−1^). (Dotted line is divining the pretreatment and post-treatment biomarker yield.) (**c**) Reactive oxygen species (ROS) production in liver tumor MPS and traditional 96-well cell culture model with DMSO and different concentrations of plant extracts (100 ng mL^−1^, 10 ng mL^−1^, 1 ng mL^−1^) at the end of the experiments or after 6 days. Both cell culture models were treated with the DMSO and extracts for 72 h after 3 days of stable cell culture. The columns exhibit the ratios of ROS produced by the treatment (plant extracts) and DMSO. (**d**) The bar graph represents the activity of superoxide dismutase (SOD) in the traditional 96-well cell culture model and liver tumor MPS after treatment with DMSO and different concentrations of plant extracts (100 ng mL^−1^, 10 ng mL^−1^, 1 ng mL^−1^) at the end of the experiments or after 6 days.

The lowest concentration of plant extract (1 ng/mL) showed a less notable effect on the urea and albumin production than the conventional cell culture models, which forecast the compromised anticancer activity results by the traditional cell culture models. ALT and AST are intracellular hepatic enzymes, and their extracellular presence indicates the underlying damage to the hepatic cells. Anticancer extracts damage the hepatocellular carcinoma cells, and the intracellular hepatic enzymes leak from the cancer cells’ damaged cell membrane [32,33]. Therefore, ALT and AST measurements were performed for liver tumor MPS (Figure 3a,b). The control experiment showed less consistent ALT and AST release than DMSO. The release of higher concentrations of ALT and AST are associated with subsequent damage to the hepatocellular carcinoma cells [34]. The plant extracts of *A. cappadocicum* increased ALT and AST in the liver tumor MPS, representing the plant extracts’ efficacy against the hepatocellular carcinoma cells.

### 3.3. Comparative Analysis of Cell Viability

**Figure 4:**
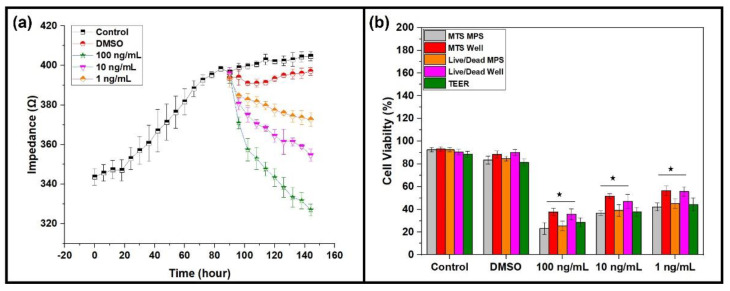
(**a**) Real-time TEER measurement graph represents the comparative impedance to different concentrations of extracts (100 ng mL^−1^, 10 ng mL^−1^, 1 ng mL^−1^) and DMSO in the liver tumor MPS. The increasing values of TEER are exhibiting the cell growth, differentiation, cellular tight junction formation, and a compact monolayer tissue formation until 72 h. DMSO and different concentrations of extracts (100 ng mL^−1^, 10 ng mL^−1^, 1 ng mL^−1^) were perfused in the liver tumor MPS on day 3, which led to a drop in TEER caused by cell-to-cell tight junction disruption. (**b**) The graph shows the comparative percentage cell viability of the traditional 96-well cell culture model and liver tumor MPS. The cell viability was calculated after the 6 six days of cell culture in both settings, e.g., 96-well cell culture model and liver tumor MPS. A *p*-value ≤ 0.05 was considered significant versus the lowest value and denoted by *.

Cellular health consists of ubiquitous mechanisms in several pathophysiological operations, including cancers. Therefore, estimating cell viability is vital to assess cells’ physical integrity and evaluate the impact of various assaults, such as toxins, mechanical factors, and chemical compounds [35,36]. Several biochemical and optical assays have been designed and employed to estimate cell viability. However, a considerable difference rests in their results owing to several uncontrolled factors, such as dye penetration, non-standardized protocols, and chances of personal error [10,37,38]. Hence, two different cell viability assessment assays have been performed in this study, as shown in Figure 4b. In addition, the impedimetric relative cell index was measured using the TEER values described previously [10]. The difference in cell viability exhibited by three different cell viability assessment methods highlights the importance of a standardized cell viability method. However, the TEER-based impedimetric relative cell index represents superiority over MTS and live/dead assay, as both show only absolute cell viability.

### 3.4. Effects of A. cappadocicum on Liver Tumor MPS

**Figure 5:**
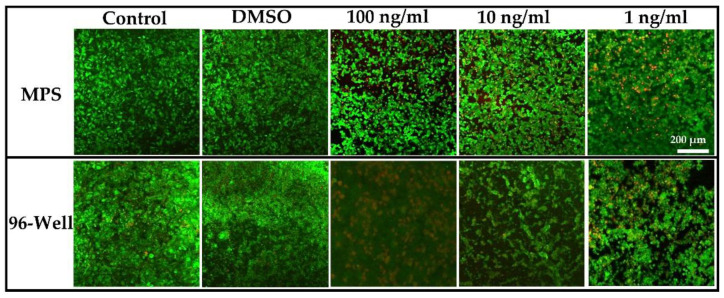
Live/dead assay confocal images on liver tumor MPS and 96-well cell culture plates without any treatment (control), DMSO, and after treatment with plant extracts (100 ng mL^−1^, 10 ng mL^−1^, 1 ng mL^−1)^. Results exhibit the cell viability after 72 h of incubation at 37 °C. Scale bars represent 200 µm.

Microfluidics offers shear stress for the optimum growth of the cells within a microenvironment by applying mechanical forces against the apical cell membrane. Fluidic mechanical stress significantly improves cell physiology, differentiation, cytokine production, and responses to pharmaceutical agents, such as drugs and plant extracts [39,40,41,42,43,44]. A similar phenomenon was observed in the present study. Liver tumor MPS biomarker yield was found to be at least onefold increased compared to the conventional 96-well cell culture plate model. Hepatocellular carcinoma cells showed better cellular viability with a liver tumor MPS than the traditional cell culture model (Figure 5). The extracts of *A. cappadocicum* were applied to both cell culture models, such as the liver tumor MPS and conventional cell culture model. However, the responses of the hepatocellular carcinoma cells were found to be more prominent in liver tumor MPS. The traditional cell culture model showed less anticancer activity of the plant extracts than the liver tumor MPS. ROS are essential for cellular signaling, but they must be in equilibrium to conserve the characteristic cellular function in a tumor niche. Therefore, plant extracts’ ROS release and antioxidant capacity are allied determinants of their anticancer activities [45,46]. The image analysis results of ROS estimation suggest more ROS found within the liver tumor MPS than the conventional cell culture model (Figure 3c). SOD is the cell’s gatekeeper for regulating ROS release, and their expression is a crucial element for cell survival by inhibiting tumor growth and metastasis [47,48]. SOD expression in the traditional cell culture model was found more than in the liver tumor MPS, which suggests higher ROS release in the liver tumor MPS (Figure 3d).

## 4. Conclusions

In conclusion, the present study proposed a novel method of anticancer activity assessment of plant extracts or derivatives by using the methanolic extracts of *Acer cappadocicum Gled*. We strongly believe that the proposed method can be used with all plant extracts or derivatives for anticancer drug discovery and development. Microfluidics-based liver tumor MPS was found to be better than the traditional cell culture model for quantifying the response of hepatocellular carcinoma cells against the plant extracts. The *Acer cappadocicum Gled* extracts showed better anticancer activity in liver tumor MPS than the traditional 96-well-based cell culture model. Hence, these extracts can be investigated further for pure compound isolation for anticancer drug development. However, the cell viability and biomarker release were significantly altered in the conventional cell culture model compared to the micro-engineered controlled liver tumor MPS. Moreover, the conventional methods of anticancer activity assessment can lead to false-negative results or present the lower anticancer efficacy of the candidate compound. In contrast, TEER-sensor-embedded liver tumor MPS offers a noninvasive and robust method to estimate plant extract efficacy compared to the traditional bioassays. Hence, our liver tumor MPS and real-time monitoring system can be employed for studying reliable anticancer activity assessment of plant-based extracts and compounds before in vivo studies.

## Data Availability

The data supporting this study are available from the corresponding authors upon reasonable request.
